# Flavonoids from *Agrimonia pilosa* Ledeb: Free Radical Scavenging and DNA Oxidative Damage Protection Activities and Analysis of Bioactivity-Structure Relationship Based on Molecular and Electronic Structures

**DOI:** 10.3390/molecules22030195

**Published:** 2017-02-26

**Authors:** Liancai Zhu, Jinqiu Chen, Jun Tan, Xi Liu, Bochu Wang

**Affiliations:** 1 Key Laboratory of Biorheological Science and Technology (Chongqing University), Ministry of Education, College of Bioengineering, Chongqing University, Chongqing 400030, China; JinqiuChen@126.com (J.C.); wangbc2000@126.com (B.W.); 2 Chongqing Key Laboratory of Medicinal Resources in the Three Gorges Reservoir Region, School of Biological & Chemical engineering, Chongqing University of Education, Chongqing 400067, China; liuxi900626@126.com

**Keywords:** *Agrimonia pilosa* Ledeb, DNA oxidative damage, flavonoids, free radical scavenging activity, bioactivity-structure relationship

## Abstract

To clarify the substantial basis of the excellent antioxidant capacity of *Agrimonia pilosa* Ledeb. Fourteen flavonoids were isolated and identified from *Agrimonia pilosa* Ledeb, seven of which have notable DPPH radical scavenging activities, i.e., catechin, luteolin, quercetin, quercitrin, hyperoside, rutin, luteolin-7-*O*-β-glucoside with IC_50_ values of 5.06, 7.29, 4.36, 7.12, 6.34, 6.36 and 8.12 µM, respectively. The DNA nicking assay showed that five flavonoids from *Agrimonia pilosa* Ledeb—taxifolin, catechin, hyperoside, quercitrin and rutin—have good protective activity against DNA oxidative damage. Further, we analyzed the bioactivity-structure relationship of these 14 flavonoids by applying quantum theory. According to their O-H bond dissociation enthalpy (BDE), C ring’s spin density and stable molecular structure, the relationship between their structures and radical scavenging capacities was evaluated and clarified. We found that among flavonoid aglycones from *Agrimonia pilosa* Ledeb, the O-H BDE of quercetin is lowest with the values of 69.02 and the O-H BDE of apigenin is highest with the values of 79.77. It is interesting that the O-H BDE value of isovitexin (78.55) with glycoside at C-6 position is lower than that of its aglycone (79.77) and vitexin (99.20) with glycoside at C-8 position. Further analysis indicated that the glycosidation of flavonoids at C-6 in the A-ring makes a more uniform distribution of spin density and improves the stability of free radicals leading to the increase in antioxidant capacity. Flavonoids with good antioxidant capacity might contribute to the pharmacological effects of *Agrimonia pilosa* Ledeb.

## 1. Introduction

The normal metabolism of all oxygen and exogenous factors could generate reactive oxygen species (ROS), such as superoxide anion radicals (O_2_^−^·), hydroxyl radical species (·OH), singlet oxygen (^1^O_2_), and hydrogen peroxide (H_2_O_2_) [1,2]. ROS are a class of highly reactive molecules that can attack a wide range of molecules found in living cells, such as protein, lipids, and nucleic acids, leading to tissue oxidative damages [3,4]. This oxidative damage has been confirmed to be involved in occurrence and development of several chronic human diseases, namely cardiovascular diseases, neurodegenerative diseases, rheumatism, diabetes mellitus and cancer [5,6,7,8]. Based on the lack of effective therapies for most chronic diseases and the usefulness of foods rich in antioxidants in the prevention of the mentioned diseases, there is a growing interest in searching for natural antioxidants from vegetables, fruits, tea, spice, and medical herbs [9,10,11].

*Agrimonia pilosa* Ledeb, a traditional Chinese medicine and a wild vegetable for tonic function, belongs to Rosaceae. As the most common *Agrimonia* spp. in China, it is used to treat tumor, and blood, gastrointestinal, genitourinary, and gynecological diseases in Chinese traditional medicine [12,13]. Our previous study found that the aqueous extract from *Agrimonia pilosa* Ledeb, especially its AcOEt-soluble fraction and *n*-BuOH soluble fraction, was rich in phenolic compounds and exhibited high antioxidant activities [14]. In the present paper, we isolated several flavonoids from the Ethyl acetate-soluble fraction (ESF) and evaluated their radical scavenging activities by using DPPH scavenging assay and protective activities on DNA oxidative damage by using DNA nicking assay to elicit the active principles present in ESF from *Agrimonia pilosa* Ledeb. Further, we studied the bioactivity-structure relationship by applying quantum chemistry theory. To date, this is the first report on free radical-scavenging effects of pure compounds obtained from the title plant. Especially, we reported firstly that glycosylation at C-6 could enhance antioxidant activity compared to the corresponding aglycones, which would offer theoretical guidance for the design and the development of antioxidants.

## 2. Results and Discussion

### 2.1. Isolation and Identification of Flavonoids from Agrimonia pilosa Ledeb

Based on the lowering blood sugar activity, more than twenty compounds had been isolation and identification, such as daucosterol, taxifolin, quercetin-3-*O*-β-d-glucopyranoside, tormentic acid, apigenin, luteolin, etc. from agrimony [15,16]. Kato et al. isolated 12 compounds from EtOAc fraction of MeOH extract from *Agrimonia pilosa* Ledeb and their α-glucosidase inhibitory activity were evaluated [17].

**Figure 1:**
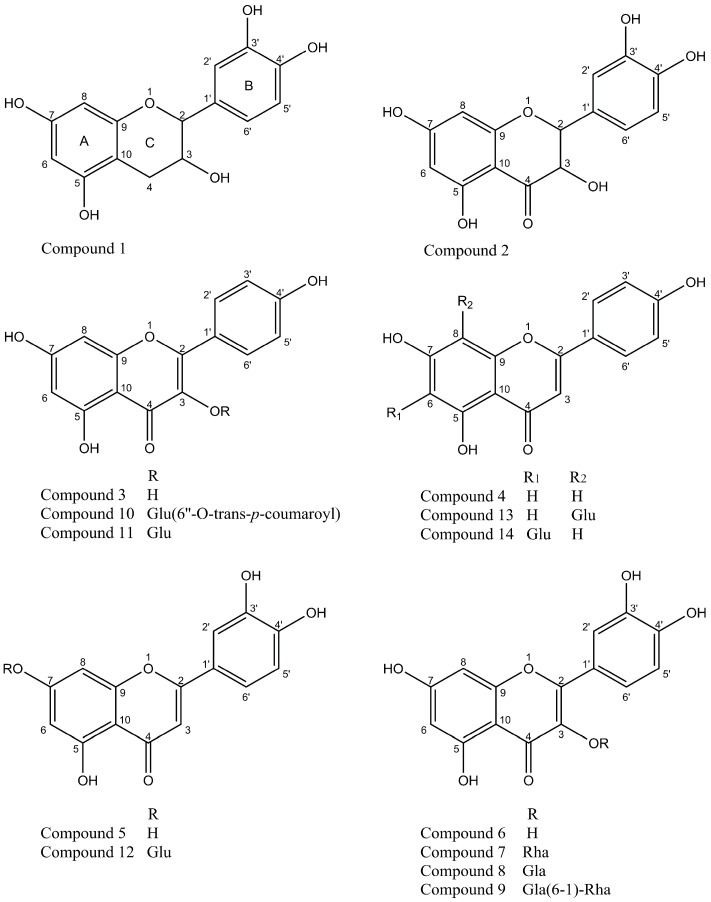
Chemical structures of flavonoids from *Agrimonia pilosa* Ledeb. **1**. catechin, **2**. taxifolin, **3**. kaempferol, **4**. apigenin, **5**. luteolin, **6**. quercetin, **7**. quercitrin, **8**. hyperoside, **9**. rutin, **10**. tiliroside, **11**. kaempferol-3-*O*-glucoside **12**. luteolin-7-*O*-β-glucoside, **13**. vitexin, **14**. isovitexin.

In our previous study, *Agrimonia pilosa* Ledeb was found to be abundant in substance with antioxidative activity [18] and 55% EtOH is the best solvent to extract the antioxidative substance (data not shown). The 55% EtOH extract of *Agrimonia pilosa* Ledeb was partitioned successively with petroleum ether, AcOEt, and *n*-BuOH and H_2_O. The EtOAc soluble fraction showed the strongest scavenging effect on the DPPH radical with an IC_50_ value of 4.88 µg/mL. Repeated chromatographic purification of EtOAc soluble fraction led to the isolation of 14 compounds. Through the interpretation of their spectra data (see supporting information) and comparison with those reported in the literature, these compounds were identified as **1**, catechin; **2**, taxifolin; **3**, kaempferol; **4**, apigenin; **5**, luteolin; **6**, quercetin; **7**, quercitrin; **8**, hyperoside; **9**, rutin; **10**, tiliroside; **11**, kaempferol-3-*O*-glucoside **12**, luteolin-7-*O*-β-glucoside; **13**,vitexin; **14**, isovitexin [19,20]. Their chemical structures were shown in Figure 1. To the best of our knowledge, this is the first report for compounds **13** and **14** to be isolated from *Agrimonia pilosa* Ledeb.

### 2.2. DPPH Radical Scavenging Activity of Flavonoids from Agrimonia pilosa Ledeb

To clarify which compounds isolated are responsible for the excellent antioxidant activity of *Agrimonia pilosa* Ledeb, the antioxidant ability of each compound was evaluated by DPPH radical scavenging activity assay, and compared with the well-known reference antioxidants, vitamin C and BHT (butylated hydroxytoluene). As shown in Table 1, the active compounds **1**, **5**–**9** and **12**, which all possessed the catechol group in ring B, exhibit potent anti-oxidant activities against DPPH with IC_50_ values of 5.06, 7.29, 4.36, 7.12, 6.34, 6.36 and 8.12 µM, respectively, whereas the positive control, vitamin C and BHT showed DPPH scavenging activities with IC_50_ values of 14.62 and 17.67 µM. The DPPH radical scavenging activity of kaempferol (IC_50_ = 16.09 µM) with the 3-hydroxyl group in the C-ring is equivalent to BHT. However, the compounds **4**, **10**, **11**, **13** and **14** which all have not the catechol group in the B-ring and the 3-hydroxyl group in the C-ring show weak radical scavenging activity with IC_50_ values larger than 100 μM.

Additionally, it is implied that the antioxidant activity of flavonoid glycoside is correlate with the position of the flavonoids glycosylation. Glycosylation sharply brings down the antioxidant activity if -OH is the active group, e.g., kaempferol-3-*O*-glucoside. On the other hand, when -OH is not the active group, glycosylation hardly affects the antioxidant activity, e.g., luteolin-7-*O*-β-glucoside. The same phenomenon was found in trans-resveratrol and trans-piceid [21].

In DPPH radical scavenging activity assay, the antioxidants reduce the very stable DPPH radical to a yellow-colored compound, diphenylpicrylhydrazine, and the extent of the reaction depends on the hydrogen-donating capacity of the antioxidants. An antioxidant candidate that proves promising in the DPPH antioxidant assay would provide an optimistic scaffold for prospective in vivo studies [22]. Thus, this assay has been extensively used for screening antioxidants from fruit and vegetable juices or natural extracts [23,24,25].

### 2.3. Protective Effect against DNA Oxidative Damage

**Figure 2:**
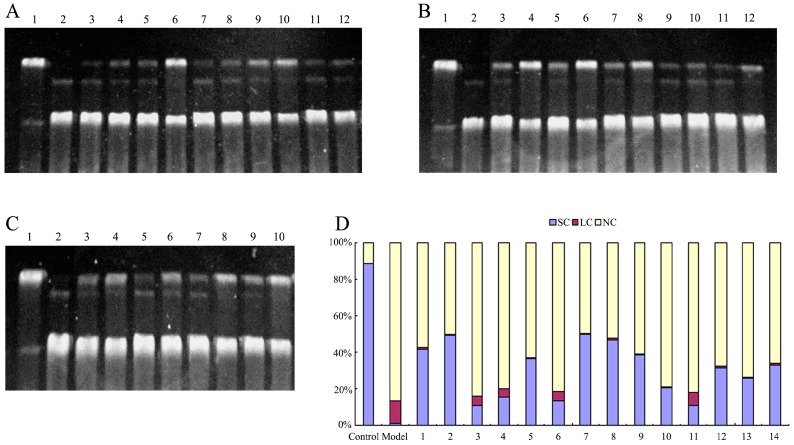
Protective effect of flavonoids from *Agrimonia pilosa* Ledeb against pBR322 DNA cleavage induced by oxidative injury. (**A**) Agarose gel electrophoresis. Lane 1: DNA control; lane 2: Model, H_2_O_2_, 100 mM; lanes 3, 4: Quercetin, 0.1 mM, 1.0 mM; lanes 5, 6: Taxifolin, 0.1 mM, 1.0 mM; lanes 7, 8: Kaempferol, 0.1 mM, 1.0 mM; lanes 9, 10: Luteolin, 0.1 mM, 1.0 mM; lanes 11, 12: Apigenin, 0.1 mM, 1.0 mM; (**B**) Agarose gel electrophoresis. Lane 1: DNA control; lane 2: Model, H_2_O_2_, 100 mM; lanes 3, 4: Hyperoside, 0.1 mM, 1.0 mM; lanes 5, 6: Quercitrin, 0.1 mM, 1.0 mM; lanes 7, 8: rutin, 0.1 mM, 1.0 mM; lanes 9, 10: Kaempferol-3-*O*-glu, 0.1 mM, 1.0 mM; lanes 11, 12: Tiliroside, 0.1 mM, 1.0 mM; (**C**) Agarose gel electrophoresis. Lane 1: DNA control; lane 2: Model, H_2_O_2_, 100 mM; lanes 3, 4: Luteolin-7-*O*-glu, 0.1 mM, 1.0 mM; lanes 5, 6: Vitexin, 0.1 mM, 1.0 mM; lanes 7, 8: Isovitexin, 0.1 mM, 1.0 mM; lanes 9, 10: catechin, 0.1 mM, 1.0 mM; (**D**) Analysis results of agarose gel electrophoresis (at the concentration of 1.0 mM).

Supercoiled plasmid pBR322 DNA, a closed double-stranded DNA molecule, has been widely used to screen the active substance protecting DNA from oxidative damage [26,27]. Plasmid pBR322 DNA exists three forms: complete pBR322 DNA, usually in supercoiled conformation (supercoiled conformation (SC)), open-loop notch type (nicked circular form (NC)), in which an incision occurs on a chain, and line form (linear form (LC)), in which the two chains break at the same position. Three forms of pBR322 DNA have completely different mobility in the gel electrophoresis: supercoiled DNA (SC) is the most tightly structured and migrates fastest, open-loop notch-type DNA (NC) migrates slowest due to its loose structure, and linear DNA (LC) is somewhere in between. Therefore, the protective effects of samples on DNA oxidative damage could be studied through observing migration position and the quantity of these three forms DNA on gel. The experimental results were shown in Figure 2.

As shown in Figure 2, SC DNA was found as the main form making up 88.2% of pBR322 DNA in the control group without H_2_O_2_, while NC DNA (87.0%) was the main form existing in the model group in which ·OH was generated to fragment DNA by using UV irradiation on H_2_O_2_.

Flavonoids from *Agrimonia pilosa* Ledeb all have protective activity on the oxidative fragment of DNA with a concentration-dependent manner. Among flavonoid aglycones from *Agrimonia pilosa* Ledeb, taxifolin exhibited the strongest efficiency, followed by catechin, luteolin, apigenin, quercetin, and kaempferol. At the presence of 1 mM taxifolin, SC percentage remains 48.8%. Three glycosides of quercetin, namely hyperoside, quercitrin and rutin, have higher protective effects than their flavonoid aglycone against the oxidative fragment of DNA with SC percentage of 49.5%, 46.7% and 38.5% at 1 mM, respectively. The protective effect of tiliroside on DNA damage is higher than that of kaempferol, while the protective effect of kaempferol-3-*O*-glu is similar to that of kaempferol. The protective effect of luteolin-7-*O*-glucoside is slightly lower than that of luteolin. Vitexin and isovitexin, as the *C*-glycosides of apigenin, have a higher protective effect on oxidative damage of DNA than their flavonoid aglycone.

### 2.4. Analysis of Bioactivity-Structure Relationship Based on the Electronic Structure

Using quantum chemistry calculation methods, the O-H bond dissociation enthalpy (BDE) and free radical spin density of the 14 flavonoids from *Agrimonia pilosa* Ledeb were studied. The relationship between the free radical scavenging activity of flavonoids and their electronic structure was illustrated from the perspective of quantum chemistry theory.

The O-H BDEs of flavonoids from *Agrimonia pilosa* Ledeb were calculated as listed in Table 2. In non-polar solvents, the antioxidants scavenge free radicals primarily by hydrogen abstraction reaction. The lower the BED of O-H is, the easier the hydrogen abstraction of the compound is, and the stronger the activity of the antioxidant is [28]. For the compound with several phenolic hydroxyl groups, its free radical scavenging activity mainly depends on the phenolic hydroxyl group with lowest BDE. From Table 2, we found that among flavonoid aglycones from *Agrimonia pilosa* Ledeb, the O-H BDE of quercetin is lowest and the O-H BDE of apigenin is highest. According to the values of BDEs, the order of free radical scavenging activities is quercetin > catechin > luteolin > taxifolin > kaempferol > apigenin. Among eight flavone glycosides, the O-H BDEs of quercitrin, hyperoside, rutin and luteolin-7-*O*-glu are lower than 73.30, which showed that they have higher free radical scavenging activities than isovitexin, vitexin, kaempferol-3-*O*-glu and tiliroside with the O-H BDEs higher than 76.32. It is interesting that the O-H BDE of isovitexin with glycoside at C-6 position is lower than that of its aglycone (apigenin) and vitexin with glycoside at C-8 position, indicating that glycoside at C-6 position is helpful for free radical scavenging activity. The evaluation results of free radical scavenging activities of flavonoids from *Agrimonia pilosa* Ledeb according to the O-H BDEs were consistent with those of the DPPH scavenging assay (see Table 1).

Besides O-H BDE, the uniformity of the spin density of radicals generated is another factor relating to the antioxidant activity of compounds [29,30]. If the unpaired electrons of the radical are highly delocalized through conjugated system, the radical energy will significantly reduce and the radical becomes stable. Semi-quinone radical has strong antioxidant activity, in which uniform spin density distribution is easy to generate.

**Figure 3:**
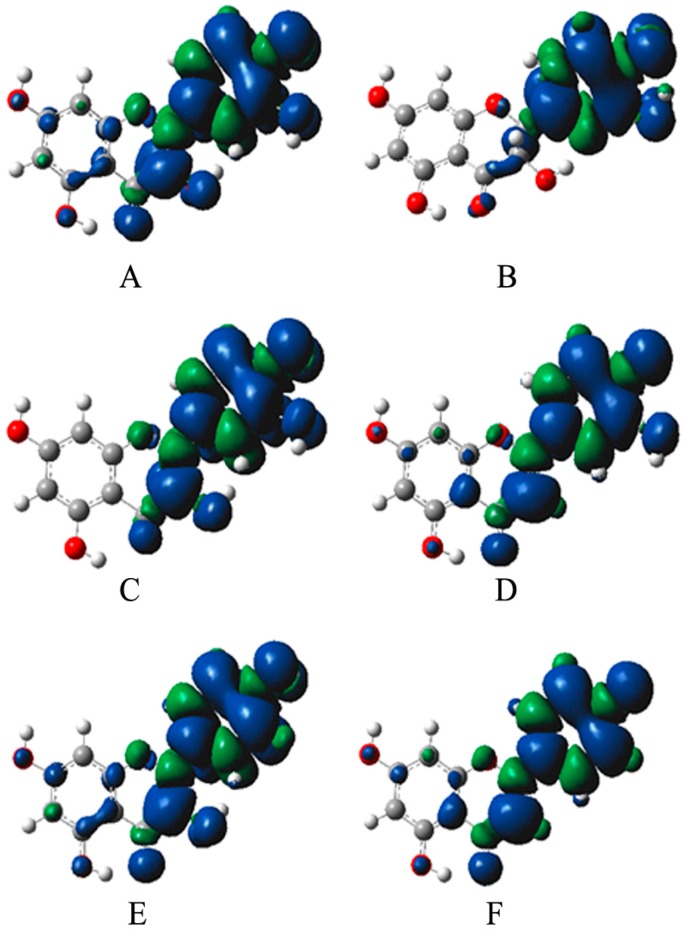
The 4′-radical spin density sketch map of six flavonoid aglycones. (**A**) Quercetin; (**B**) Taxifolin; (**C**) Catechin (4′-radical); (**D**) Luteolin; (**E**) Kaempferol; (**F**) Apigenin.

The spin density sketch maps of six flavonoid aglycones from *Agrimonia pilosa* Ledeb were shown in Figure 3. The C-ring’s spin density of compounds was listed in Table 3, Table 4, Table 5 and Table 6. The spin density of 4′-O is closely related to antioxidant activity. We found that 4′-Os of luteolin, quercetin, quercitrin and rutin have low spin density, indicating these compounds have good radical scavenging activities, while 4′-Os of kaempferol-3-*O*-glu, tiliroside, apigenin, apigenin-6-glu, apigenin-8-glu have high spin density, implying that these compounds possess weak radical scavenging activities. 

According to the BDE values, the C-ring’s spin density and the stable molecular structure, the following conclusions could be deduced: (1) The presence of 3-OH, the ortho-hydroxy in the B-ring, 2,3-double bond and 4-carbonyl contribute to a more balanced distribution of spin density, reducing the BDE of O-H in the B-ring and increasing free radical scavenging activity of compounds; (2) Glycosylation of 3-OH in flavonol increases the dihedral angle between the B-ring and C-ring, resulting in an increase in spin density imbalance and the BDE of O-H in the B-ring, and thus reduces the free radical scavenging activity of compounds; (3) Glycosidation of flavonoids at C-6 in the A-ring makes a more uniform distribution of spin density and improves the stability of free radicals; whereas glycosidation at C-8 in the A-ring increases the dihedral angle between C-ring and B-ring, resulting in reduction of the delocalization degree and the stability of free radicals. The results of theoretical calculations are consistent with those of experimental studies.

## 3. Materials and Methods

### 3.1. Chemicals

Ethidium bromide and 1,1-diphenyl-2-picrylhydrazyl were purchased from Sigma Chemical Co. (St. Louis, MO, USA). pBR322 DNA was purchased from Toyobo Co., Ltd. (Osaka, Japan). Sephadex LH-20 was purchased from GE healthcare (Uppsala, Sweden), silica gel and precoated silica gel plates were purchased from Qingdao Haiyang Chemical Co., Ltd. (Qingdao, China). All other reagents were of analytical grade.

### 3.2. Plant Material

The dried aerial parts of *Agrimonia pilosa* Ledeb were purchased from Western Medicine City in Chongqing, China.

### 3.3. Chemical Experiments

#### 3.3.1. Extraction and Isolation

Extraction of the dried aerial parts of *Agrimonia pilosa* Ledeb with EtOH and H_2_O (EtOH:H_2_O = 55:45) and fractionation of the former extract with solvent–solvent extraction were done with CHCl_3_, AcOEt, and *n*-BuOH successively. AcOEt fraction was then applied to a silica gel column (100 × 1000 mm, 200–300 mesh) using CHCl_3_–MeOH (50:1), (30:1), (10:1), (5:1) and (1:1) solvent systems, successively. Eluents were grouped according to TLC control with 10% sulfate in ethanol as color-developing agent, using (a) CHCl–MeOH–CHOOH (8:2:0.1) and (b) CHCl_3_–MeOH (10:1) solvent systems resulting in four fractions (F1, F2, F3 and F4), in which F2 and F3 shows flavonoids constituents. F2 and F3 were further chromatographed on a silica gel column (40 × 800 mm, 200–300 mesh) with CHCl_3_–MeOH–CHOOH (8:2:0.1) solvent system and a Sephadex LH-20 column (35 × 800mm) using CHCl_3_-MeOH (1:1) as eluents repeatedly to give compound 1–14.

#### 3.3.2. Structure Elucidation of the Isolated Compounds

Examination of TLC plates under UV light and after visualization by spraying reagents revealed that these compounds possess a flavonoid skeleton. Molecular weights of isolated flavonoids were analyzed by using 5050E MS (VG, UK). Structure of isolated flavonoids were elucidated by using ^1^H-NMR (500 MHz; with TMS as international standard) and ^13^C-NMR (100.2 MHz) spectroscopy in AVANCE DRX-500 NMR Spectrometers (Bruker BioSpin, Fallanden, Switzerland). The NMR spectra of compounds were taken in DMSO. Then the compounds were identified by direct comparisons of their molecular weight and spectral data (^1^H-NMR and ^13^C-NMR) with those of literature data.

### 3.4. DPPH Radical Scavenging Assay

The ability of the flavonoids to scavenge DPPH radicals was determined according to the method of Blois [31] with some modifications. Briefly, a 0.1 mL sample was mixed with 1.9 mL of 0.1 mm DPPH in EtOH. The concentration of the tested samples in the mixture was 5.0, 10.0, 20.0, 30.0, 40.0 and 50.0 μM, respectively. The blanks contained all the reagents except the compound tested or positive-control substances. After incubation for 30 min in darkness, the resulting absorbance was recorded at 517 nm. The percentage scavenging values were calculated from the absorbance of the blank (A_0_) and of the sample (A_S_) according to the following equation:

DPPH radical-scavenging activity [%] = (1 − A_S_/A_0_) × 100%, (1)

The IC_50_ (concentration for 50% DPPH radical-scavenging activity) values were determined by SPSS software. Ascorbic acid and butylated hydroxytoluene (BHT) were used as positive controls.

### 3.5. DNA Nicking Assay

DNA nicking assay was used to test the damage effect of ·OH on DNA and the protective ability of flavonoids from *Agrimonia pilosa* Ledeb on DNA oxidative damage. DNA damage was measured by the conversion of supercoiled pBR322 plasmid DNA to nicked circular DNA and linear DNA forms. Tris-HCl buffer (50 mmol/L pH 7.2), the sample solution, pBR322 DNA (200 ng/reaction), and H_2_O_2_ were added into a 0.5 mL EP tube, diluted to a total volume of 10 μL with distilled water, and mixed. After the mixture was irradiated for 10 min by UV lamp, 2 μL of loading buffer was added to terminate the reaction. Then the mixture was subjected to electrophoresis in a horizontal slab gel apparatus and 1 × TAE buffer, which was performed at 75 V for 1.5 h. The gel was stained with a solution of 0.5 μg/mL ethidium bromide for 30 min, followed by destaining in water. Agarose gel electrophoresis of plasmid DNA was visualized by photographing the fluorescence of intercalated ethidium bromide under a UV illuminator. After electrophoresis, the proportion of DNA in each form was estimated quantitatively from the intensity of the bands using Glyko BandScan software.

The control group did not contain H_2_O_2_ and the compound tested, while the model group contained H_2_O_2_ but not the compound tested.

### 3.6. Quantum Theory Study of Bioactivity-Structure Relationship

The O-H bond dissociation enthalpy (BDE) and free radical spin density were adapted to study and analyze the bioactivity-structure relationship of flavonoids from *Agrimonia pilosa* Ledeb.

Using a combination method of density functional theory proposed by Wright et al. [32], the O-H bond dissociation enthalpy (BDE) was calculated. First, the optimization molecular structure of flavonoids was obtained by AMl approach. Then, the frequency of molecular vibration was calculated at B3LYP/6-31(d) level in order to check whether a stable configuration of each molecular was obtained. Meanwhile, zero point vibrational energy (ZPVE) was calculated and corrected by using the correction factor of 0.9806. Finally, single point electronic energy (SPE) was calculated at the B3LYP/6-31G (d) level. The total energy of molecules includes single-point energy and zero-point vibrational energy corrected. For flavonoid containing glycoside, layered Oniom (B3LYP/6-31d: UHH) was used for calculation. 

The following formula was used to give the value of BDE.

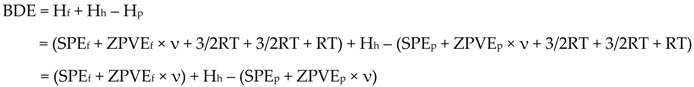
(2)

In the formula, H_p_ is the enthalpy of the parent molecule; H_h_, the enthalpy of hydrogen atom, is −0.49792 Hartree; H_f_ is the enthalpy of free radicals generated from precursor molecule losing a hydrogen atom. Temperature was set at the standard temperature. The correction factor ν = 0.9806. All calculations were performed with the Gaussian 03 program [33].

### 3.7. Statistical Analysis

Data were collected and expressed as the mean ± standard deviation of three independent experiments and analyzed for statistical significance from control, using the Dunnett test (SPSS 11.5 Statistics Software; SPSS, Chicago, IL, USA). The criterion for significance was set at *p* < 0.05. IC_50_ values, from the in vitro data, were calculated by regression analysis.

## 4. Conclusions

We isolated and identified 14 flavonoids from *Agrimonia pilosa* Ledeb. Catechin, taxifolin, luteolin, quercetin, quercitrin, hyperoside, rutin and luteolin-7-*O*-β-glucoside have notable radical scavenging activities and are responsible for the excellent antioxidant activity of *Agrimonia pilosa* Ledeb according to DPPH radical scavenging assay and quantum theory study. It is worth noting that glycosidation of flavonoids at C-6 in the A-ring makes a more uniform distribution of spin density and improves the stability of free radicals, leading to the increase of antioxidant capacity. In addition, catechin, taxifolin, quercitrin, hyperoside and rutin exhibit a good protective effect on DNA oxidative damage. It is implied that these flavonoids with good antioxidant capacity might contribute to some of the pharmacological and nutrient effects of *Agrimonia pilosa* Ledeb. Some researches should be launched to develop function foods or drugs for diseases relating to oxidative damage.

## Figures and Tables

**Table 1:** DPPH scavenging activity of flavonoids from *Agrimonia pilosa* Ledeb with IC_50_^1,2^ (μM) values.

Number	Compound	IC_50_ (μM)
**1**	catechin	5.06 ± 0.08
**2**	taxifolin	11.98 ± 0.12
**3**	kaempferol	16.09 ± 0.10
**4**	apigenin	>200
**5**	luteolin	7.29 ± 0.09
**6**	quercetin	4.36 ± 0.10
**7**	quercitrin	7.12 ± 0.11
**8**	hyperoside	6.34 ± 0.10
**9**	rutin	6.36 ± 0.12
**10**	tiliroside	>200
**11**	kaempferol-3-*O*-glucoside	>200
**12**	luteolin-7-*O*-β-glucoside	8.12 ± 0.14
**13**	vitexin	>200
**14**	isovitexin	122.83 ± 0.20
**15**	vitamin C	14.62 ± 0.15
**16**	BHT	17.67 ± 0.11

^1^ Means of three experiments. ^2^ Values obtained from regression lines. IC_50_ is defined as the concentration sufficient to obtain 50% of maximum radical scavenging.

**Table 2:** The calculated O-H bond dissociation enthalpy (BDE) of flavonoids from *Agrimonia pilosa* Ledeb (Kcal/mol, 1 atm, 298 K).

Compounds	BDE (Kcal/mol)				
	3-OH	5-OH	7-OH	3′-OH	4′-OH
Quercetin	78.95	102.70	83.37	71.86	69.02
Taxifolin	96.05	100.78	84.62	80.17	72.64
Catechin	78.44	77.86	80.20	77.13	69.47
Luteolin	-	102.77	83.97	80.10	72.44
Kaempferol	78.33	102.72	83.37	-	74.73
Apigenin	-	97.78	83.98	-	79.77
Isovitexin	-	80.68	79.70	-	78.55
Vitexin	-	101.61	101.52	-	99.20
Kaempferol-3-*O*-glu	-	89.93	81.22	-	78.69
Luteolin-7-*O*-glu	-	87.05	-	80.45	73.30
Tiliroside	-	77.96	79.49	-	76.32
Rutin	-	89.25	85.38	84.13	73.04
Quercitrin	-	91.22	82.36	80.10	69.89
Hyperoside	-	92.18	84.71	75.99	71.11

**Table 3:** The C-ring’s spin density of apigenin, vitexin and isovitexin.

Compound	4′-O	6′-C	5′-C	4′-C	3′-C	2′-C	1′-C
Apigenin	0.380083	−0.169869	0.303191	−0.113358	0.274517	−0.163938	0.382626
vitexin	0.373175	−0.168035	0.300523	−0.108348	0.267208	−0.160770	0.382844
isovitexin	0.396971	−0.166433	0.301826	−0.112689	0.290139	−0.164036	0.383058

**Table 4:** The C-ring’s spin density of luteolin and luteolin-7-*O*-glu.

Compound	4′-O	6′-C	5′-C	4′-C	3′-C	2′-C	1′-C	3′-O
Luteolin	0.338555	−0.076213	0.184145	−0.015767	0.236716	−0.135202	0.316232	0.083901
luteolin-7-*O*-glu	0.351547	−0.131706	0.247106	−0.080224	0.247929	−0.146586	0.345674	0.069776

**Table 5:** The C-ring’s spin density of kaempferol, kaempferol-3-*O*-Glu and tiliroside.

Compound	4′-O	6′-C	5′-C	4′-C	3′-C	2′-C	1′-C
Kaempferol	0.362436	−0.151734	0.263024	−0.104162	0.278057	−0.152102	0.351079
kaempferol-3-*O*-Glu	0.387743	−0.161792	0.289969	−0.108207	0.290449	−0.162656	0.380708
Tiliroside	0.366781	−0.159347	0.281211	−0.103501	0.274765	−0.158234	0.373993

**Table 6:** The C-ring’s spin density of quercetin, quercitrin, hyperoside and rutin.

Compound	4′-O	6′-C	5′-C	4′-C	3′-C	2′-C	1′-C	3′-O
Quercetin	0.316394	−0.063693	0.165670	−0.012205	0.246853	−0.131286	0.299326	0.088008
Quercitrin	0.334737	−0.097927	0.206668	−0.093886	0.245379	−0.142259	0.308832	0.097475
Hyperoside	0.323551	−0.060549	0.164803	−0.010714	0.254078	−0.129996	0.297435	0.090597
Rutin	0.325680	−0.070428	0.164597	−0.016466	0.251577	−0.129888	0.302433	0.090028

## Supplementary Materials

Supplementary materials can be accessed at: http://www.mdpi.com/1420-3049/22/3/195/s1. Table S1: ^1^H-NMR data of isolated flavonoids from *Agrimonia pilosa* Ledeb, Table S2: ^1^^3^C-NMR data of isolated flavonoids from *Agrimonia pilosa* Ledeb, Table S3: ESI-MS (*m*/*z*) data of isolated flavonoids from *Agrimonia pilosa* Ledeb.
