# Supplementary material for: Flavonoids from Agrimonia pilosa Ledeb: Free Radical Scavenging and DNA Oxidative Damage Protection Activities and Analysis of Bioactivity-Structure Relationship Based on Molecular and Electronic Structures
Source: Molecules. 2017 Feb 26;22(3):195. doi: 10.3390/molecules22030195 (PMC6155215; doi:10.3390/molecules22030195)
Supplement: Supplementary file 1 [file molecules-22-00195-s001.pdf]

Supplementary Materials: Flavonoids from *Agrimonia pilosa* Ledeb: Free Radical Scavenging and DNA Oxidative Damage Protection Activities and Analysis of Bioactivity-Structure Relationship Based on Molecular and Electronic Structures

Liancai Zhu, Jinqiu Chen, Jun Tan, Xi Liu and Bochu Wang

Table S1. ^{1}H-NMR data of isolated flavonoids from *Agrimonia pilosa* Ledeb.

Position	Catechin	Taxifolin	Kaempferol	Apigenin	Luteolin	Quercetin	Quercitrin	Hyperoside	Rutin	Tiliroside	Kaempferol-3-O-glucoside	Luteolin-7-O-β-glucoside	Vitexin	Isovitexin
2	δ4. 48 (1 H, d, 7. 5)	δ4.98(d，11.0)												
3	3. 81 (1 H, m)	δ4.50(dd，11.0，6.0)；	10.12 (s, OH)	6.76（s）	6.68（s）	10.03 (s, OH)						6.75（1H, s）	6.28（s）	6.48（s）
4	δ2. 64 (1 H, dd, 16. 0，5.0 Hz) 、 2. 34(1 H, dd, 16. 0，8. 0)	δ5.82（d，6.0，OH）												
5			12.49(s, OH)	12.95(s, OH)	12.99 (s, OH)	12.41 (s, OH)	12.69 (s, OH)	12.71 (s, OH)	12.69 (s, OH)	12.59(s, OH)	12.51(s, OH)	13.01 (s, OH)	13.17(s, OH)	13.15(s, OH)
6	5. 69 (1 H, d, 2. 0)	11.90 (s, OH)	6.44（d, 2.0）	6.47（d, 2.0）	6.19 (d, 2.0)	6.20 (d, 2.0)	6.25 (d, 2.0)	6.20 (d, 2.0)	6.20 (d, 2.0)	6.40（d, 2.0）	6.43（d, 2.0）	6.44 (d, 2.0)	6.78（s）	
7		δ5.88(d，1.5)	10.80 (s, OH)			10.61 (s, OH)	10.89 (s, OH)	10.78 (s, OH)	10.77 (s, OH)	10.86 (s, OH)	10.78 (s, OH)		10.84 (s, OH)	10.79 (s, OH)
8	5.89 (1 H, d, 2. 0)	11.00 (s, OH)	6.19（d, 1.5）	6.17（d, 2.0）	6.45 (d, 2.0)	6.44 (d, 2.0)	6.43 (d, 2.0)	6.42 (d, 2.0)	6.40 (d, 2.0)	6.16（d, 2.0）	6.19（d, 2.0）	6.64 (d, 2.0)		6.76（s）
9		δ5.93(d，1.5)												
10														
1′														
2′	δ6.73 (1 H, d, 2. 0)	6.76(q)	8.04 (d, 8.5)	7.91 (d, 9.0)	7.43 (d 2.0)	7.35 (d 2.0)	7.35 (d 2.0)	7.35 (d 2.0)	7.61 (d, 2.0)	7.99 (dd, 2.0, 9.0)	8.03 (d, 8.5)	7.44 (d 2.0)	8.02 (d, 8.5)	7.99 (d, 8.5)
3′		9.15 (s,	6.92(d,	6.91(d,		9.26 (s,	9.38 (s,	9.36 (s,	9.62 (s,	6.86(dd,	6.91(d,		6.88 (d,	6.86 (d,

		OH)	8.5)	8.5)		OH)	OH)	OH)	OH)	2.0, 9.0)	8.5)		9.0)	9.0)
4′		9.09 (s, OH)	9.41 (s, OH)			9..52 (s, OH)	9.74 (s, OH)	9.69 (s, OH)	9.13(s, OH)	10.17 (s, OH)	9.39 (s, OH)		10.35 (s, OH)	10.41 (s, OH)
5′	6.67 (1H, d, 8.5)	6.76(q)	6.92 (d, 8.5)	6.91 (d, 8.5)	6.89 (d, 8.5)	6.91 (d, 8.5)	6.91 (d, 8.5)	6.87 (d, 8.5)	6.83 (d, 8.5)	6.86(dd, 2.0, 9.0)	6.91(d, 8.5)	6.91 (d, 8.5)	6.88 (d, 9.0)	6.86 (d, 9.0)
6′	6.59 (1 H, dd, 8. 5,2.0)	6.89 (s)	8.04(d, 8.5)	7.91(d, 9.0)	7.40 (dd, 8.5, 2.0)	7.29 (dd, 8.5, 2.0)	7.29 (dd, 8.5, 2.0)	7.58 (dd, 8.5, 2.0)	7.59（dd, 2.0, 8.5）	7.99(dd, 2.0, 9.0)	8.03(d, 8.5)	7.47(dd, 8.5, 2.0)	8.02 (d, 8.5)	7.99(d, 8.5)
1″														
2 "														
3 "														
4 "														
5 "														
6 "							0.83 (d, 6.0)							
1'''														
2'''										6.11(d, 16.0)				
3'''										7.34(d, 16.0)				
4'''														
5''''										7.37(d, 8.5)				
6'''										6.79(d, 8.5)				
7'''										10.03 (s, OH)				
8'''										6.79(d, 8.5)				
9'''										7.37(d, 8.5)				

Table S2. ^{13}C-NMR data of isolated flavonoids from *Agrimonia pilosa* Ledeb.

Position	Catechin	Taxifolin	Kaempferol	Apigenin	Luteolin	Quercetin	Quercitrin	Hyperoside	Rutin	Tiliroside	Kaempferol-3-O-glucoside	Luteolin-7-O-β-glucoside	Vitexin	Isovitexin
2	81.05	83.26	146.21	164.42	163.87	146.84	156.54	156.46	156.78	156.34	155.98	163.88	163.9	163.7
3	66.37	71.74	135.65	102.85	102.85	135.82	134.32	133.52	133.54	133.06	133.03	102.93	102.41	102.62
4	27.89	197.94	175.89	181.77	181.64	175.64	177.84	177.56	177.63	177.39	177.84	181.53	182.05	181.95
5	156.52	163.49	160.69	161.48	161.45	160.43	161.39	161.28	161.39	161.14	156.56	160.81	161.09	161.16

6	95.18	96.22	98.18	98.95	98.8	98.08	98.78	98.71	98.88	98.75	98.21	99.59	98.1	108.1
7	156.25	166.98	163.87	163.78	164.11	163.36	164.26	164.13	164.15	164.14	163.78	163.02	162.53	163.45
8	93.92	95.19	93.46	94.06	93.82	93.31	93.71	93.38	93.73	93.65	93.49	94.71	104.57	93.87
9	155.42	162.74	156.16	157.38	157.27	156.29	157.39	156.38	156.56	156.43	161.03	156.81	155.96	156.09
10	99.132	100.66	103.02	103.65	103.67	102.36	104.19	104.05	104.26	103.87	103.14	104.92	104.01	103.87
1′	130.66	128.2	121.65	121.2	118.97	121.25	121.22	121.43	121.77	120.76	121.37	119.07	121.58	121.08
2′	114.57	115.49	129.49	128.52	113.34	115.13	115.56	115.26	115.37	130.8	129.26	113.02	128.92	128.53
3′	144.9	145.13	115.43	116.04	145.71	145.17	145.27	144.91	144.93	115.74	115.36	145.16	115.77	116.03
4′	144.9	145.96	159.18	161.24	149.68	147.45	148.5	148.38	148.55	159.97	159.64	149.09	160.35	160.85
5′	115.16	115.96	115.43	116.04	115.99	115.46	115.77	116.16	116.46	115.74	115.36	115.61	115.77	116.03
6′	118.52	119.68	129.492	128.52	120.07	120.07	120.87	121.31	121.36	130.8	129.26	119.83	128.92	128.53
1″							101.89	102.13	101.33	100.96	101.36	100.03	78.63	79.32
2 "							70.47	71.41	74.33	76.22	74.23	73.26	73.34	73.63
3 "							70.67	73.36	76.54	74.22	76.49	76.89	70.8	71.06
4 "							71.3	68.09	70.16	69.96	70.08	69.91	70.51	70.76
5 "							70.15	75.75	75.98	74.11	77.26	77.34	81.8	81.63
6 "							17.57	60.62	67.12	62.95	61.21	61.23	61.26	61.56
1'''									100.88	166.15				
2'''									70.49	113.63				
3'''									70.72	144.57				
4'''									71.88	124.92				
5''''									69.41	130.12				
6'''									17.78	115.07				
7'''										159.77				
8'''										115.07				
9'''										130.12				

Table S3. ESI-MS (*m*/*z*) data of isolated flavonoids from *Agrimonia pilosa* Ledeb.

	Catechin	Taxifolin	Kaempferol	Apigenin	Luteolin	Quercetin	Quercitrin	Hyperoside	Rutin	Tiliroside	Kaempferol-3-O-glucoside	Luteolin-7-O-β-glucoside	Vitexin	Isovitexin
$[M + H]^+$	291	305	287	271	287	303	449	465	611	595	449	449	433	433
